# Validity and reliability of the Arabic version of knowledge, attitudes, and practices toward COVID-19 preventative behaviors (KAP COVID-19)

**DOI:** 10.3389/fpubh.2023.1131843

**Published:** 2023-12-14

**Authors:** Eman Bajamal, Mona Alanazi

**Affiliations:** ^1^College of Nursing, King Saud bin Abdulaziz University for Health Sciences, Jeddah, Saudi Arabia; ^2^King Abdullah International Medical Research Center, Riyadh, Saudi Arabia; ^3^Ministry of the National Guard Health Affairs, Riyadh, Saudi Arabia; ^4^College of Nursing, King Saud bin Abdulaziz University for Health Sciences, Riyadh, Saudi Arabia

**Keywords:** scale, psychometric, knowledge, attitude, practice, Saudi Arabia

## Abstract

**Background:**

Even though the innovation of COVID-19 vaccination effectively protects against the virus, practicing preventative behaviors is still essential. However, public adherence to preventive behaviors relies mainly on the individuals’ knowledge, attitudes, and practices (KAP) toward COVID-19 preventative behaviors. Therefore, it is crucial to evaluate these aspects. Nevertheless, there is no validated scale that assesses KAP toward COVID-19 preventative behaviors. To evaluate the psychometric properties of the Arabic version of knowledge, attitudes, and practices toward coronavirus disease 2019 (COVID-19) preventative behaviors.

**Methods:**

A quantitative descriptive cross-sectional design was used to develop and evaluate the psychometric properties of KAP through specific stages: item development, face, content, convergent and construct validity evaluation, and internal consistency. Data were collected online through “google forms” from June 2020 to July 2020. Both exploratory factor analysis (EFA) and confirmatory factor analysis (CFA) were used to assess the construct validity.

**Results:**

A total of 1,363 participants completed the scale. The overall Cronbach’s alpha was 0.83, indicating good internal consistency. Exploratory factor analysis supported structural validity for all the scale items with KMO 0.78, 0.60, and 0.81, respectively, and Bartlett’s Test of Sphericity = (*p* < 0.05). Convergent validity was confirmed by a moderate correlation between the KAP scale items, knowledge ranging from *r* = 0.11 (*p* < 0.01) to *r* = 0.62 (*p* < 0.01), attitude ranging from *r* = 0.158 (*p* < 0.01) to *r* = 0.584 (*p* < 0.01), and practice ranging from *r* = 0.383 (*p* < 0.01) to *r* = 0.774 (*p* < 0.01).

**Conclusion:**

The psychometric properties of the scale indicate that the KAP is a valid and reliable scale that can be utilized to evaluate the level of KAP toward COVID-19 preventative behaviors among the population in Saudi Arabia.

## 1 Introduction

Coronavirus disease 2019 (COVID-19) is an infectious respiratory disease caused by the Coronavirus virus, which contributes to severe health conditions and death cases worldwide (1). On 11 March 2020, due to the seriousness of this virus and its rapid transitions, the World Health Organization (WHO) announced that COVID-19 is a global pandemic and called for prevention actions (2). Therefore, COVID-19 has become a public health crisis threatening all countries worldwide (3). COVID-19 has affected 230 countries; to date 20 August2022, and the number of confirmed cases has exceeded 600 million, with almost 6.5 million fatal cases worldwide (4). In Saudi Arabia, over 800,000 individuals have been infected with COVID-19, with over 9,000 deaths (5). Moreover, during the current stage of this pandemic, when vaccines are available and quite efficient, many countries no longer instruct their population to practice preventative behaviors, including wearing masks outdoors, avoiding crowded places, and maintaining social distancing. In fact, it is still essential to maintain these preventative behaviors alongside the vaccination to end this pandemic and to prevent further COVID-19 waves (6).

The only way to slow the virus’s spread is to prevent its transmission among the population through vaccination, awareness, and preventative measures (7). Even though vaccination is integral in fighting the COVID-19 pandemic, the WHO highlighted the importance of other prevention behaviors, including social distancing, hand hygiene, self-isolation when sick, and avoiding crowded places (8). These prevention behaviors have played an integral part in the last year in minimizing the spread of COVID-19 and, therefore, must be continued in all countries with outbreaks and ongoing transmission, including Saudi Arabia.

In Saudi Arabia, during the pandemic, actions and protection policies at the national level were highly encouraged and promoted through national media. For example, mandating masks in public places and advising individuals to practice hand hygiene principles (9). Adhering to these actions and protection policies is still one of the critical measures for ending the pandemic.

While the innovation of COVID-19 vaccination is effective in stopping the transmission of the virus and concerning the dynamics of COVID-19 prevention and management (e.g., changes in regulations of wearing masks outdoors), it is crucial to keep the preventative behaviors, such as wearing masks, hand hygiene, and cough etiquette, especially as the COVID-19 waves did not end (6).

This study is the first psychometric study to develop and analyze the psychometric properties of measuring knowledge, attitudes, and practices toward COVID-19 preventive behaviors. The literature review findings revealed that numerous questionnaires had been used in previous studies conducted in Saudi Arabia (10–14). However, none of them are a validated scale to measure KAP toward COVID-19 preventive behaviors. In fact, the questionnaire used by Wajid et al. 2021, Wajid et al. 2022, Al-Rawi et al. and Alqahtani et al. was adapted and modified from a previous study that intended to measure the effectiveness of Cholera vaccination campaigns (10–12, 14). Moreover, other studies that developed surveys to measure KAP targeted specific groups, not the general population, such as paramedics, healthcare workers, and nursing students (15–17). There are only two studies that targeted the general population in Saudi Arabia. However, the first study did not report their questionnaire’s content or face validity (18). In addition, the second study reported that their questionnaire was developed based only on the Saudi MOH guidelines. Also, the knowledge domain had only four items, which may not represent the knowledge domain (19). Furthermore, there were some studies exist on the validation of cognitive behavior scales during COVID-19 (20, 21), on COVID-19 and mental effects, (22) COVID-19 and smoking (23), insomnia and social media use (24), and satisfaction with online learning (25, 26). For instance a previous study by Fares et al. developed and validated a psychometrically reliable instrument to assess psychological distress during the COVID-19 pandemic across Arab countries (20). Similarly, Aljaberi et al. in 2022 validated Coronavirus Disease 2019 (COVID-19) -induced psychological distress, including a 22-item impact of Event Scale-Revised (IES-R) and concluded that the tools were reliable screening instruments for measuring COVID-19 related distress (21). Therefore, there is a possibility that these questions do not represent the whole aspects of the COVID-19 knowledge domain.

Therefore, the population’s knowledge, attitude, and practice (KAP) toward preventive behaviors against this disease are vital in ending COVID-19. Moreover, examining the KAP of the population during a pandemic is highly imperative and is considered one of the central pillars of pandemic control (8). Therefore, a valid and reliable scale is urgently needed for this aim. Due to the lack of a valid and reliable scale in Saudi Arabia, this study aimed to develop and evaluate the psychometric properties of an Arabic version instrument about knowledge, attitude, and practice (KAP) toward COVID-19 preventive behaviors among the population in Saudi Arabia.

## 2 Methods

### 2.1 Design, sample, and setting

A quantitative descriptive cross-sectional design was used to describe the level of KAP toward COVID-19 preventative behaviors. Data were collected online through the “google forms” platform from June 2020 to July 2020. The sample size was estimated by the G Power software, which allows sample size analysis and high-precision power and computes the power values for sample size, effect size, and alpha levels. The sample included 1,363 participants to achieve the power of 95% with an effect size of 0.1, a margin of error of 5%, and missing data was estimated as 15%. Participants were enrolled in this research if they were (1) 18 years and older, (2) able to read and write Arabic, (3) living in Saudi Arabia, (4) have internet, and (5) have basic technology skills to answer the scale. Participants were excluded if they did not meet the inclusion criteria.

### 2.2 Procedure

The data collection process started after obtaining IRB approval from the King Abdullah International Medical Research Center (KAIMRC). On the first page of the data collection form, the researcher explained the subjects, the aims of the study, and the expectations of participation. Also, the researchers clarified that there is no potential risk associated with participation in this study, and the participants can withdraw from the research at any time without penalty. Moreover, no identifiers or personal information were collected or stored to maintain the privacy and confidentiality of the participants, including participants’ names, IDs, and others. Then the participants were asked if they were willing to participate in this study and consented if they agreed. All this information was sent through the electronic app “WhatsApp,” and then a snowballing method was used to send the invitation to other people.

### 2.3 Measures

To ensure the rigorousness of the scale, the scale development process was carried out through the following procedures: item development, face validity evaluation, content validity evaluation, and internal consistency.

### 2.4 Item development

The researchers specify three concepts of interest: Knowledge (K), Attitude (A), and Practice (P) of the population toward COVID-19 preventive behaviors among adults in Saudi Arabia. To create items assessing the three concepts, the researchers conducted a literature review and searched the current clinical and community management guidelines for COVID-19 by the Saudi Ministry of Health (MOH) (27). The literature search was guided by the following questions: (1) What is the necessary information about COVID-19 prevention; (2) What attitude toward COVID-19 preventative behaviors should the population have?; and (3) What should the population practice to prevent the spread of COVID-19? Moreover, the researchers independently searched the COVID-19 prevention guidelines developed by the WHO, CDC, and Saudi MOH (7, 8, 27).

The researchers independently recognized pertinent areas for measuring knowledge, attitudes, and practices toward COVID-19 preventive behaviors, and then compared and merged the findings. The bibliographic databases used to conduct the literature search included CINHAL, PubMed, Scopus, PsycINFO, Web of Science (ISI), and Science Direct. The scale involved an interface page and two main sections. The interface page included the title, objective of the study, information on participants’ privacy, and the electronic consent form to agree to participate in this study. The first section consisted of socio-demographic characteristics, including age, gender, marital status, educational status, occupational status, place of current residence, and medical history. The second section of the scale consisted of three parts, which are the following: (1) questions of general knowledge regarding COVID-19, (2) attitude, and (3) practice towards COVID-19 preventative behaviors. The first part, general knowledge regarding COVID-19, has 16 items with “true,” “false,” and “I do not know” answers. The “I do not know” answer was added to differentiate incorrect knowledge from lack of knowledge and minimize the probability of a respondent opting for the correct answer by chance. These items cover COVID-19’s etiology, transmission mode, clinical symptoms, risk factors, isolation, preventive measures, and treatment. The second part, attitude towards COVID-19 preventative behaviors, has six items using the “agree,” “disagree,” and “sometimes “scale. In this section, the participants were asked about their attitudes toward COVID-19 preventative behaviors, including if they (a) need to visit the health care facility when they feel sick and have some of the COVID-19 symptoms, (b) have confidence that they can win the battle against the COVID-19 virus, (c) need to isolate themselves from others if they experienced any symptoms of COVID-19, (d) know the importance of washing their hands in protecting them from COVID-19, (e) believe that social distancing keeps them safe from COVID-19, whenever they are outside their home, and (f) agree that following the guidelines from the Ministry of Health will prevent the spread of COVID-19. Lastly, the third part included the practice toward COVID-19 preventative behaviors and has five items with “Yes,” “No,” and “Sometimes” answers. In this section, the participants were asked about their practice toward COVID-19 preventative behaviors, including (a) wearing a mask when they go out, (b) visiting any crowded places, (c) washing their hands with soap and water for 40 s, or rubbing them with an alcohol-based sanitizer for 20 s, (d) visiting social event involving a large number of people, and (e) avoiding shaking hands.

Similar to previous studies the scores were calculated by assigning, a zero score for the incorrect or “I do not know” answers, and correct answers were given 1 point (28–34). The total score on the scale ranges from 0 to 16. A higher score indicates a higher knowledge of COVID-19. For the second part, attitude towards COVID-19 preventative behaviors, which has six items using the “agree,” “disagree,” and “sometimes “scale, 1 point was given to “disagree”; 2 points were given to “sometimes”; and 3 points were given to “agree” (28). Therefore, the total score ranges from 6 to 18, with a higher score indicating a higher practice toward COVID-19 preventative behaviors. For the last part, practice towards COVID-19 preventative behaviors, which has five items with “Yes,” “No,” and “Sometimes” answers, 1 point was given to the favorable answer, and 0 points were given to the unfavorable or sometimes answers (29). Therefore, the total score ranges from 0 to 5, with a higher score indicating a higher positive attitude toward COVID-19 preventative behaviors. The first draft of the scale was developed in English and then translated to Arabic using a backward translation approach. Discrepancies between both languages were ensured after consultation with bilingual researchers. The scale was reviewed, validated, and pilot-tested to evaluate its face and content validity, and internal consistency reliability.

### 2.5 Face validity evaluation

The researchers used the face validity approach to obtain feedback and evaluate the ease of understanding for each item (28). A convenience sample of 100 individuals from the population of Saudi Arabia was invited to participate in the pilot study. Based on the inclusion and exclusion criteria, participants who agreed and gave electronic informed consent were included. Therefore, a total of 70 participants were included in the pilot study and were asked to provide feedback on each item.

### 2.6 Content validity evaluation

To ensure the relevancy of the scale’s content, the Delphi method was utilized, and the items were reviewed based on the content validity indices (CVIs) for both individual items (I-CVI) and the total scale (S-CVI) (35). Moreover, based on Lynn’s recommendation, it is essential to have a minimum of six individuals to have a panel of subject matter experts to review the content representatives and the relevancy of the items (35). Therefore, the initial draft of KAP toward the COVID-19 preventative behaviors scale was sent to a heterogeneous group of 10 individuals who are either experts in scale development or have experience working in government-designated COVID-19 care centers. Eight of the invited experts agreed to participate in reviewing the scale’s content and consented electronically. The initial draft of KAP was sent through the “google form” to their emails. They were asked to assess each item’s importance to the scale’s aim and determine the relevance of the items to the content domains of the scale. To illustrate, they were asked to evaluate how well each item reflected the main three concepts of KAP on a four-point Likert scale. The scoring method used is as follows: 1 = not relevant, 2 = somewhat relevant, 3 = relevant, and 4 = highly relevant (20). They were also recommended to provide feedback about each item and the general constructions of the initial scale.

The content validity of the KAP scale was evaluated by examining the inter-rater reliability or agreement regarding all items of the scale (36). According to Wongpakaran et al. an instrument with inter-rater reliability greater than 75% is considered an acceptable scale (37). As demonstrated in Table 1, the scale had an acceptable inter-rater reliability coefficient above 75%. Moreover, according to Polit and Beck (2004), the content validity index (CVI) can be assessed by measuring the item content validity index (I-CVI) and the scale content validity index (S-CVI). An I-CVI of 0.83 and an S-CVI of 0.80 or above are considered acceptable (38). As demonstrated in Table 1, the scale had acceptable I-CVI and S-CVI above 0.80 and 0.83. The internal consistency was assessed to determine the consistency of the items throughout the scale by calculating Cronbach’s alpha for the scale (39). As shown in Table 1, the internal consistency of the scale ranged from 0.85 to 0.95, with the highest Cronbach’s *α* for the practice of COVID-19 preventative behaviors. The KAP scale had good reliability based on criteria for “acceptable internal consistency” (39).

**Table 1:** Content validity for the KAP scale.

Scale	Inter-rater reliability coefficient (%)	I-CVI	S-CVI/UA	Cronbach’s *α*
Knowledge towards COVID_19	87	1.00	1.00	0.85
Attitude towards COVID_19	95	1.00	1.00	0.90
Practice towards COVID_19	100	1.00	1.00	0.95

### 2.7 Ethical considerations

This study was reviewed and approved by the research committee at KSAU-HS, college of nursing, Riyadh region, and KAIMRC (Ref No: RYD-21-419812-45587). Invited participants were fully informed about the purpose and the expectation of participation in the study. The participants’ privacy and confidentiality were assured. Electronic informed consent was obtained from each participant prior to participating in the study. Participants were informed that their participation was voluntary and that they could withdraw at any time without penalty.

### 2.8 Statistical analysis

Statistical Package for the Social Sciences computer software (SPSS for Mac, Version 23.0; IBM, 2015) was used to analyze the data. To describe study variables, descriptive statistics were calculated on all variables of interest, including means, standard deviations, frequencies, and percentages. Cronbach’s alpha and McDonald’s Omega coefficients were used to test internal consistency (38, 40). A scale with a Cronbach’s alpha greater than 0.70 has good internal consistency (41, 42) and with McDonald’s Omega coefficients greater than 0.70 has good internal consistency (40). Intraclass correlation coefficient (ICC) was used to test the stability of the scale (43–45). Item-total correlations were used to identify any items not correlating well (<0.20) with the overall scale before examining validity (46). Exploratory factor analysis and confirmatory factor analysis were used to assess the construct validity. According to Williams et al. Kaiser-Meyer-Olkin (KMO) with 0.5 is considered acceptable (47), and Bartlett’s Test of Sphericity should be significant (*p* < 0.05). Root Mean Square Error of Approximation (RMSEA) < 0.08 is considered absolute fit; and Comparative Fit Index (CFI) > 0.90, Normed Fit Index (NFI) > 0.90 is considered incremental fit (48, 49). The Pearson correlation coefficient was used to assess the convergent validity of the scale. Statistical significance was based on the standard alpha level of 0.05.

## 3 Results

### 3.1 Participants demographics

A total of 1,363 participants completed the electronic survey. The mean COVID-19 knowledge score was 12.34 (SD = 2.02), and the overall accuracy rate for the knowledge test was 77.13%. The mean attitude score for COVID-19 was 17.61 (SD = 1.14), indicating positive attitudes. The mean score for practice was 3.47 (SD = 2), indicating an acceptable level of practice. Of the total sample, the majority of the participants were between the ages of 30 and 39 (46.5%), female participants (58.9%), married (80.6%), had a baccalaureate degree of education (56.9%), full-time employed (66.3%), not working in the health field (80.9%), and living in the central region of Saudi Arabia (67.2%). Refer to Table 2 for more details of the participants’ socio-demographic characteristics.

**Table 2:** Socio-demographic characteristics of the participants (*n* = 1,363).

	Characteristics	M	SD	*n*	(%)
Knowledge		12.34	2.02		
Attitude		15.47	2.49		
Practice		3.19	1.92		
Age	18–29			308	22.6
	30–39			634	46.5
	40–49			348	25.5
	>50			73	5.4
Residency	Central region			916	67.2
	Eastern region			211	15.5
	Western region			121	8.9
	Southern region			66	4.8
	Northern region			49	3.6
Gender	Male			560	41.1
	Female			803	58.9
Marital status	Never married			213	15.6
	Married			1,100	80.6
	Widowed			12	0.9
	Divorced			38	2.8
Educational status	Uneducated			82	6
	Elementary school			21	1.5
	Middle school			31	2.3
	High school			285	20.9
	Baccalaureate degree			776	56.9
	Graduate degree			168	12.3
Employment status	Unemployed but physically able to work			274	20
	Physically unable to work			15	1.1
	Employed Part-Time			82	6
	Employed Full Time			904	66
	Retired			88	6.5
Work in the health sector	Yes			261	19.1
	No			1,102	80.8

### 3.2 Psychometric properties

#### 3.2.1 Reliability

The scale had three concepts to be measured or assessed among the participants as follows: knowledge (16 items), attitude (6 items), and practice toward COVID-19 preventative behaviors (5 items).

As demonstrated in Table 3, Cronbach’s alpha coefficient was 0.83 and McDonald’s Omega coefficient was 0.63 for the overall KAP scale of 27 items. In addition, Cronbach’s alpha coefficients were 0.75 for the 16-item of the Knowledge domain, 0.75 for the 6-item of the attitude domain, and 0.86 for the 5-item of the practice domain. In addition, McDonald’s Omega coefficients were 0.73 for the 16-item of the knowledge domain, 0.74 for the 6-item of the attitude domain, and 0.86 for the 5-item of the practice domain. For the knowledge of COVID-19 preventative behaviors, Item-total correlation coefficients ranged from 0.004 to 0.62. There were several items that had small item-total correlation coefficients (<0.20), which were items 3, 5, 6, 7, 8, 9, 10, 11, 12, 13, 14, 15, and 16. For the attitude towards COVID-19 preventative behaviors, the item-total correlation ranged from 0.15–0.58. There was only one item that had a small item-total correlation coefficient (<0.20) which was item 5 (Social distancing keeps you safe from COVID-19, whenever you are outside your home). For the practice of COVID-19 preventative behaviors, the item-total correlation ranged from 0.38 to 0.77.

**Table 3:** Reliability of the scale.

KAP Scale	M	SD	Cronbach’s *a*	McDonald’s Omega
Knowledge Towards COVID-19	0.76	0.34	0.75	0.73
Attitude towards COVID-19	2.57	0.61	0.75	0.74
Practice towards COVID-19	0.63	0.47	0.86	0.86
Overall KAP Scale	1.13	0.42	0.83	0.63

To test the stability of the KAP scale, ICC was 0.71 for the overall KAP scale of 27 items with a 95% CI of [0.69, 0.73]. Also, the ICC was calculated separately for each domain as follows: 0.75 for the 16-item of the knowledge domain with a 95% CI of [0.73, 0.77]; 0.75 for the 6-item of the attitude domain with a 95% CI of [0.73, 0.77]; and 0.86 for the 5-item of the practice domain with a 95% CI of [0.85, 0.87].

#### 3.2.2 Convergent validity

The Pearson Correlation coefficient was used to test the convergent validity of the KAP scale. As shown in Table 4, the 16 items of knowledge about COVID-19 were moderately to strongly correlated (50) with each other, with coefficients ranging from *r* = 0.11 (*p* < 0.01) to *r* = 0.62 (*p* < 0.01). In addition, as shown in Table 5, the 6 items of the attitude towards COVID-19 preventative behaviors were moderately to strongly correlated (50) with each other with coefficients ranging from *r* = 0.158 (*p* < 0.01) to *r* = 0.584 (*p* < 0.01). Also, as shown in Table 6, the 6 items of the practice towards COVID-19 preventative behaviors were moderately to strongly correlated (50) with each other with coefficients ranging from *r* = 0.383 (*p* < 0.01) to *r* = 0.774 (*p* < 0.01).

**Table 4:** Pearson’s product–moment correlations for the knowledge towards COVID-19 (*N* = 1,363).

Variable	1	2	3	4	5	6	7	8	9	10	11	12	13	14	15	16
1 - Question 1	–															
2 - Question 2	0.36^**^	–														
3 - Question 3	0.03	−0.01	–													
4 - Question 4	0.21^**^	0.22^**^	0.53^**^	–												
5 - Question 5	0.15^**^	0.07^**^	0.46^**^	0.58^**^	–											
6 - Question 6	0.11^**^	0.07^**^	0.44^**^	0.39^**^	0.40^**^	–										
7 - Question 7	0.00	0.17**	0.11**	0.26**	0.27**	0.18**	–									
8 - Question 8	0.28**	0.26**	0.14**	0.36**	0.34**	0.22**	0.02	–								
9 - Question 9	0.04	0.07**	0.17**	0.26**	0.35**	0.15**	0.07**	0.39**	–							
10 - Question 10	0.21**	0.22**	−0.01	0.26**	0.30*	0.05*	0.20**	0.33**	0.18**	–						
11 - Question 11	0.09**	0.15**	−0.00	0.08**	0.15**	0.15**	0.19**	0.06*	−0.11**	0.27**	–					
12 - Question 12	−0.00	−0.03	−0.01	0.07**	0.12**	0.04	−0.08**	0.23**	0.30**	0.00	0.03	–				
13 - Question 13	0.15**	0.12**	0.29**	0.41**	0.47**	0.25**	0.19**	0.25**	0.43**	0.23**	0.13**	0.21**	–			
14 - Question 14	0.12**	0.10**	0.28**	0.40**	0.46**	0.29**	0.14**	0.26**	0.40**	0.13**	0.09**	0.32**	0.62**	–		
15 - Question 15	0.11**	0.16**	0.20**	0.28**	0.39**	0.16**	−0.01	0.54**	0.34**	0.16**	0.15**	0.29**	0.46**	0.45**	–	
16 - Question 16	−0.07**	−0.07**	0.18**	0.27**	0.25**	0.39**	0.12**	0.15**	0.13**	0.11**	0.26**	0.15**	0.23**	0.23**	0.31**	–

*p < 0.05 and **p < 0.01.

**Table 5:** Pearson’s product-moment correlations for the attitude towards COVID-19 preventative behaviors (*N* = 1,363).

Variable	1	2	3	4	5	6
1 - Question 1	–					
2 - Question 2	0.300^*^	–				
3 - Question 3	0.200^*^	0.378^*^	–			
4 - Question 4	0.299^*^	0.584^*^	0.321^*^	–		
5 - Question 5	0.319^*^	0.223^*^	0.337^*^	0.158^*^	–	
6 - Question 6	0.466^*^	0.241^*^	0.202^*^	0.514^*^	0.526^*^	–

*p < 0.05 and **p < 0.01.

**Table 6:** Pearson’s product-moment correlations for the practice toward COVID-19 preventative behaviors (*N* = 1,363).

Variable	1	2	3	4	5
1 - Question 1	–				
2 - Question 2	0.459*	–			
3 - Question 3	0.601*	0.383*	–		
4 - Question 4	0.515*	0.529*	0.581*	–	
5 - Question 5	0.624*	0.490*	0.619*	0.774*	–

*p < 0.05 and **p < 0.01.

#### 3.2.3 Construct validity

An exploratory factors analysis with Varimax rotation was conducted to assess the factorial structure of the KAP scale. The results of Bartlett’s test indicated that there were significant correlations among the 16 items of knowledge towards COVID-19 preventative behaviors [*χ*^2^(120) = 6447.04, *p* < 0.0001], and the Kaiser-Meyer-Olkin (KMO) measures of sampling adequacy was 0.78. Also, the results of Bartlett’s test of attitude towards COVID-19 preventative behaviors indicated that there were significant correlations among the 6 items [*χ*^2^(15) = 2314.35, *p* < 0.0001], and the KMO measures of sampling adequacy was 0.60. In addition, the results of Bartlett’s test of practice towards COVID-19 preventative behaviors indicated that there were significant correlations among the 5 items [*χ*^2^(10) = 3346.57, *p* < 0.0001], and the KMO measures of sampling adequacy was 0.81, indicating that the factor analysis can yield distinct and reliable factors for KAP scale (47).

#### 3.2.4 Confirmatory factor analysis

A confirmatory factor analysis was conducted also to test the factorial structure of the KAP scale. Results of the confirmatory factor analysis model indicated that the six-factor structure of knowledge towards COVID-19 preventative behaviors with fit indices [*x*^2^(104) = 2581.67, *p* < 0.0001, RMSEA = 0.06] with 90% CI [0.05–0.08], CFI = 0.87, NFI = 0.98, ECVI = 1.94, AIC = 2645.67. Moreover, the results of the confirmatory factor analysis model indicated that the two-factor structure of attitude towards COVID-19 preventative behaviors with fit indices [*x*^2^(9) = 765.45, *p* < 0.0001, RMSEA = 0.07] with 90% CI [0.05–0.08], CFI = 0.90, NFI = 0.67, ECVI = 0.58, AIC = 789.45. Also, the results of the confirmatory factor analysis model indicated that a one-factor structure of practice towards COVID-19 preventative behaviors with fit indices [*x*^2^(5) = 170.77, *p* < 0.0001, RMSEA = 0.06] with 90% CI [0.05–0.08], CFI = 0.95, NFI = 0.94, ECVI = 0.140, AIC = 190.77.

## 4 Discussion

To our knowledge, this is the first study rigorously developed and evaluated the psychometric properties of KAP COVID-19 in a sample of the population in Saudi Arabia. It is urgently needed to have a valid and reliable scale to measure KAP toward COVID-19 preventative behaviors. Regardless of COVID-19, communicable diseases are expected in today’s world. Indeed, fast transmissions of any new viruses may cause future pandemics. With a valid and reliable scale, researchers, healthcare providers, and stakeholders will be able to assess the requirements of intervention, and they will be able to assess the effectiveness of their utilized strategies to prevent future pandemics or further waves of COVID-19. The results of this study indicated that the KAP scale consists of 27 items designated to assess knowledge (16 items), attitude (6 items), and practice (5 items) toward COVID-19 preventative behaviors. To ensure the rigorousness of the scale, the scale development process started with item development, face validity, content validity, and psychometric propriety testing.

An intensive literature review and search of the current international and national COVID-19 protection guidelines were utilized to develop the items of the scale. Therefore, a total of 27 items were generated to investigate the three main domains of the scale: knowledge, attitude, and practice. After that, a pilot study was carried out to assess the face validity of the tool and to obtain feedback from a sample of the target population. Therefore, a convenience sample of 70 individuals from the population in Saudi Arabia was recruited to provide the researchers with feedback on the easy understanding of items. Followed by the content validity assessment to ensure the items’ relevancy and all eras of the three main domains were covered. Thus, the scale was evaluated among eight experts who have experience in scale development and COVID-19 protection behaviors.

Three main domains were investigated in this scale: knowledge, attitude, and practice toward COVID-19 preventative behaviors. The results indicate that the Cronbach’s alpha coefficient was 0.75 for the 16 items of knowledge, with item-total correlation coefficients ranging from 0.004 to 0.62. There were several items that had a small item-total correlation coefficient (<0.20), which were items 3, 5, 6, 7, 8, 9, 10, 11, 12, 13, 14, 15, and 16. It is worth mentioning that these items should remain on the scales based on the experts’ recommendations and the literature review. Moreover, similar items were included in previous studies among various populations (51–53). In addition, Cronbach’s alpha coefficient was 0.75 for the 6-item attitude, with item-total correlation ranging from 0.15–0.58. There was only one item with a small item-total correlation coefficient (<0.20) which was item 5. This item should also remain on the scale for the same reasons as it was included in a previous research study (54). This study’s result pertaining to item-total correlation coefficients for these two domains is in agreement with Park’s work, which developed and validated a tool to measure KAP toward COVID-19 prevention. According to a recent study, there were 13 items related to the knowledge domain which had a relatively lower item-total correlation coefficient value than was expected. However, Park recommended retaining those 13 items because they were determined by an extensive literature review and based on ten experts’ recommendations. Lastly, the Cronbach’s alpha coefficient was 0.86 for the 5-item of practice toward COVID-19 prevention, with item-total correlation ranging from 0.38 to 77 (51–55). This study’s findings parallel previous studies that developed and validated the KAP COVID-19 scale (51, 54). In addition to the reliability, the validity of the scale was also ensured by assessing its content and construct validity. The content validity of the KAP scale was evaluated by determining the inter-rater reliability and CVI (I-CVI and S-CVI). Based on the results of the current study, the KAP scale demonstrated an acceptable inter-rater reliability coefficient and acceptable CVI levels for all three main domains. Moreover, an expert evaluation with a value of CVI of more than 0.70 highlights that this scale is suitable for measuring KAP toward COVID-19 preventative behaviors (56). This study’s results are consistent with a previous study conducted by Saefi et al. 2020 (57) where it was found that all items have a CVI > 0.80 for all domains (KAP) with an average CVI of 0.97–0.99 (56).

For construct validity, the results of the exploratory factor analysis identified that the three domains of KAP are different and reliable factors for the KAP scale (50). Also, these results are parallel to other findings in the literature. For example, Saefi et al. and Park (50, 56) found the same results in the proposed factorial structure. Therefore, the psychometric evaluation of the scale in this study provided an indication of its validity and reliability in assessing knowledge, attitudes, and practices toward COVID-19 preventative behaviors. The findings indicated acceptable validities and reliability. This study has some strengths and limitations that should be taken into consideration. This study’s strength relies on the study sample size, which included a large sample of 1,363 participants from all regions of Saudi Arabia. However, this study was dominated by married (80.6%) and educated participants (57% have a baccalaureate degree of education). Thus, the findings revealed that the participants are knowledgeable about the COVID-19 pandemic. Despite these limitations, the results of this study support the use of the KAP scale in evaluating the level of knowledge, attitude, and practice toward COVID-19 preventative behaviors. Further studies are recommended for empirical testing to confirm the constancy of KAP toward COVID-19 preventative behaviors in diverse populations.

This research has significant implications for future practice. With the temporality of the COVID-19 regulations and the absence of mandatory strategies, the number of positive cases is rising once more. The results of this study are vital to evaluate the current situation of preventative behaviors against COVID-19. Furthermore, regardless of COVID-19, having a validated tool to measure KAP related to an infectious disease is absolutely important. Also, this scale can help stakeholders to determine the level of the KAP among the general population. Moreover, there might be future outbreaks or pandemics of other viruses, this scale can aid healthcare providers and stakeholders to plan their interventions and improve their current strategies. Furthermore, this scale can be adapted and modified to meet any communicable disease characteristics.

## 5 Conclusion

The Arabic version of the knowledge, attitude, and practice of the 27-item scale of COVID-19 showed generally satisfactory psychometric properties when applied among the Saudi population. Based on our findings, it is proposed that this version can be used for various purposes related to promoting positive knowledge attitudes, and practice toward COVID-19-related health. Nevertheless, generating more psychometric data on this scale by employing it in further studies with Saudi adults would be useful. It is imperative to have a single, valid, reliable scale to measure these dimensions to end the COVID-19 pandemic. The study results indicated an acceptable level of validity and reliability of the scale.

## Data availability statement

The original contributions presented in the study are included in the article/Supplementary material, further inquiries can be directed to the corresponding author.

## Ethics statement

The studies involving humans were approved by research committee at KSAU-HS, college of nursing, Riyadh region, and KAIMRC (Ref No: RYD-21-419812-45587). The studies were conducted in accordance with the local legislation and institutional requirements. The participants provided their written informed consent to participate in this study.

## Author contributions

All authors listed have made a substantial, direct, and intellectual contribution to the work and approved it for publication.
