# Supplementary material for: Validity and reliability of the Arabic version of knowledge, attitudes, and practices toward COVID-19 preventative behaviors (KAP COVID-19)
Source: Front Public Health. 2023 Dec 14;11:1131843. doi: 10.3389/fpubh.2023.1131843 (PMC10752940; doi:10.3389/fpubh.2023.1131843)
Supplement: Supplementary file 1 [file Table_1.docx]

**Table 4**

*Pearson’s product-moment correlations for the knowledge towards COVID-19 (N = 1363)*

| Variable | 1 | 2 | 3 | 4 | 5 | 6 | 7 | 8 | 9 | 10 | 11 | 12 | 13 | 14 | 15 | 16 |
| --- | --- | --- | --- | --- | --- | --- | --- | --- | --- | --- | --- | --- | --- | --- | --- | --- |
|  |  |  |  |  |  |  |  |  |  |  |  |  |  |  |  |  |
| 1- Question 1 | — |  |  |  |  |  |  |  |  |  |  |  |  |  |  |  |
|  |  |  |  |  |  |  |  |  |  |  |  |  |  |  |  |  |
| 2- Question 2 | .36** | — |  |  |  |  |  |  |  |  |  |  |  |  |  |  |
|  |  |  |  |  |  |  |  |  |  |  |  |  |  |  |  |  |
| 3- Question 3 | .03 | -.01 | — |  |  |  |  |  |  |  |  |  |  |  |  |  |
|  |  |  |  |  |  |  |  |  |  |  |  |  |  |  |  |  |
| 4- Question 4 | .21** | .22** | .53** | — |  |  |  |  |  |  |  |  |  |  |  |  |
|  |  |  |  |  |  |  |  |  |  |  |  |  |  |  |  |  |
| 5- Question 5 | .15** | .07** | .46** | .58** | — |  |  |  |  |  |  |  |  |  |  |  |
|  |  |  |  |  |  |  |  |  |  |  |  |  |  |  |  |  |
| 6- Question 6 | .11** | .07** | .44** | .39** | .40** | — |  |  |  |  |  |  |  |  |  |  |
|  |  |  |  |  |  |  |  |  |  |  |  |  |  |  |  |  |
| 7- Question 7 | .00 | .17** | .11** | .26** | .27** | .18** | — |  |  |  |  |  |  |  |  |  |
|  |  |  |  |  |  |  |  |  |  |  |  |  |  |  |  |  |
| 8- Question 8 | .28** | .26** | .14** | .36** | .34** | .22** | .02 | — |  |  |  |  |  |  |  |  |
|  |  |  |  |  |  |  |  |  |  |  |  |  |  |  |  |  |
| 9- Question 9 | .04 | .07** | .17** | .26** | .35** | .15** | .07** | .39** | — |  |  |  |  |  |  |  |
|  |  |  |  |  |  |  |  |  |  |  |  |  |  |  |  |  |
| 10- Question 10 | .21** | .22** | -.01 | .26** | .30* | .05* | .20** | .33** | .18** | — |  |  |  |  |  |  |
|  |  |  |  |  |  |  |  |  |  |  |  |  |  |  |  |  |
| 11- Question 11 | .09** | .15** | -.00 | .08** | .15** | .15** | .19** | .06* | -.11** | .27** | — |  |  |  |  |  |
|  |  |  |  |  |  |  |  |  |  |  |  |  |  |  |  |  |
| 12- Question 12 | -.00 | -.03 | -.01 | .07** | .12** | .04 | -.08** | .23** | .30** | .00 | .03 | — |  |  |  |  |
|  |  |  |  |  |  |  |  |  |  |  |  |  |  |  |  |  |
| 13- Question 13 | .15** | .12** | .29** | .41** | .47** | .25** | .19** | .25** | .43** | .23** | .13** | .21** | — |  |  |  |
|  |  |  |  |  |  |  |  |  |  |  |  |  |  |  |  |  |
| 14- Question 14 | .12** | .10** | .28** | .40** | .46** | .29** | .14** | .26** | .40** | .13** | .09** | .32** | .62** | — |  |  |
|  |  |  |  |  |  |  |  |  |  |  |  |  |  |  |  |  |
| 15- Question 15 | .11** | .16** | .20** | .28** | .39** | .16** | -.01 | .54** | .34** | .16** | .15** | .29** | .46** | .45** | — |  |
|  |  |  |  |  |  |  |  |  |  |  |  |  |  |  |  |  |
| 16- Question 16 | -.07** | -.07** | .18** | .27** | .25** | .39** | .12** | .15** | .13** | .11** | .26** | .15** | .23** | .23** | .31** | — |
|  |  |  |  |  |  |  |  |  |  |  |  |  |  |  |  |  |

*Note: *p < .05, **p < .01.*
